# The Vesicular Intersection Layer: A Framework for Cross-Kingdom Extracellular Vesicle Signaling That May Connect Gut Dysbiosis to Skeletal Muscle Wasting in Colorectal Cancer Cachexia

**DOI:** 10.3390/cancers18030522

**Published:** 2026-02-05

**Authors:** Young-Sool Hah, Seung-Jun Lee, Jeongyun Hwang, Seung-Jin Kwag

**Affiliations:** 1 Department of Surgery, Institute of Medical Science, Gyeongsang National University College of Medicine, Jinju 52727, Republic of Korea; yshah@gnu.ac.kr; 2 Biomedical Research Institute, Gyeongsang National University Hospital, Jinju 52727, Republic of Korea; 3 Department of Convergence Medical Sciences, Gyeongsang National University, Jinju 52725, Republic of Korea; 0789zxc@gnu.ac.kr (S.-J.L.); dkdl10252@gnu.ac.kr (J.H.)

**Keywords:** colorectal cancer, cachexia, extracellular vesicles, bacterial extracellular vesicles, gut microbiome, skeletal muscle wasting, TLR4, GDF15, biomarkers, endotypes

## Abstract

Colorectal cancer (CRC) cachexia is a multifactorial, treatment-limiting syndrome characterized by progressive loss of skeletal muscle with or without loss of fat mass, accompanied by systemic inflammation, anorexia, metabolic dysregulation, and impaired treatment tolerance. Despite decades of work, cachexia remains clinically underdiagnosed and therapeutically underserved, in part because canonical models treat tumor-derived factors and host inflammatory mediators as a largely ‘host-only’ network. In parallel, CRC is strongly linked to intestinal dysbiosis, barrier disruption, and microbial translocation. Extracellular vesicles (EVs)—host small EVs, tumor-derived EVs, and bacterial extracellular vesicles (including outer membrane vesicles)—may provide a mechanistically plausible, information-dense route by which these domains could be coupled. Here, we synthesize emerging evidence suggesting that cross-kingdom EV signaling may operate as a vesicular ecosystem spanning gut lumen, mucosa, circulation, and peripheral organs. We propose the “vesicular intersection layer” as a unifying framework for how heterogeneous EV cargos converge on shared host decoding hubs (e.g., pattern-recognition receptors and stress-response pathways) to potentially contribute to muscle catabolism. We critically evaluate what is known—and what remains unproven—about EV biogenesis, trafficking, and causal mechanisms in CRC cachexia, highlight methodological constraints in microbial EV isolation and attribution, and outline minimum evidentiary standards for cross-kingdom claims. Finally, we translate the framework into actionable hypotheses for EV-informed endotyping, biomarker development (including stool EV assays), and therapeutic strategies targeting shared signaling nodes (e.g., TLR4–p38) and endocrine mediators that are predominantly soluble but may be fractionally vesicle-associated (e.g., GDF15). By reframing CRC cachexia as an emergent property of tumor–host–microbiota vesicular communication, this review provides a roadmap for mechanistic studies and clinically tractable interventions.

## Simple Summary

Colorectal cancer can cause cachexia, a syndrome of progressive weight loss and skeletal muscle wasting that limits therapy and worsens survival. Most models emphasize tumor- and host-derived inflammatory signals, but colorectal cancer is also linked to gut dysbiosis and impaired barrier function. This review explains how extracellular vesicles—small membrane-bound particles released by tumors, host cells, and gut microbes—may transport bioactive cargo across the gut–blood interface and amplify systemic inflammation. We propose a ‘vesicular intersection layer’ in which heterogeneous vesicle signals converge on shared host decoding hubs (for example, Toll-like receptor pathways) that may activate common muscle catabolic programs and could couple peripheral wasting to anorexia. Finally, we outline minimum experimental standards for cross-kingdom claims and discuss how stool- and blood-based vesicle assays could enable patient stratification and guide therapies that target shared signaling nodes.

## 1. Introduction

CRC cachexia is a high-burden clinical phenotype that reduces quality of life, increases treatment toxicity, and worsens survival. Epidemiologically, cachexia affects 50–80% of patients with advanced cancer and accounts for approximately 20% of cancer-related deaths. In colorectal cancer specifically, the prevalence of sarcopenia and cachexia at diagnosis is estimated between 39% and 48%, rising significantly with disease progression [1,2,3]. International consensus frameworks define cancer cachexia as a multifactorial syndrome characterized by ongoing skeletal muscle loss (with or without fat loss) that cannot be fully reversed by conventional nutritional support [4,5,6,7]. These definitions emphasize negative protein and energy balance, as well as the progressive nature of wasting across the disease trajectory [8,9]. Clinical practice guidelines emphasize the importance of early recognition and multimodal supportive care [10,11]. They also emphasize standardized phenotyping and harmonized reporting to enhance trial readiness and reproducibility [12,13,14,15]. Body composition and functional readouts (e.g., CT-derived SMI, grip strength, and performance status) are prognostic across solid tumors, including gastrointestinal malignancies, and cachexia can be clinically ‘masked’ by obesity (sarcopenic obesity) [16,17,18,19,20]. CRC incidence and mortality remain substantial worldwide [21,22]. In parallel, accumulating evidence links CRC to intestinal dysbiosis and chronic mucosal inflammation [23,24]. Metagenomic studies and meta-analyses have further identified reproducible CRC-associated fecal microbial signatures across independent cohorts [25,26,27]. Yet, mechanistic and clinical models of cachexia are still predominantly framed as a tumor–host signaling problem, with limited integration of the gut ecosystem. While the previous review elegantly describes the role of microbiota-derived EVs in local gut immunity and general inter-kingdom communication [28], it largely focused on intestinal homeostasis or on broad systemic inflammation. Our review distinguishes itself by specifically addressing the ‘terminal effector’ of this communication: skeletal muscle wasting. We move beyond general inflammation to propose the ‘vesicular intersection layer’—a specific framework explaining how heterogeneous vesicular cargos from the tumor and microbiome converge on shared catabolic signaling nodes (e.g., TLR4/p38) to drive the unique phenotype of CRC cachexia.

Extracellular vesicles (EVs) offer a mechanistically distinct bridge between these domains. EVs package proteins, lipids, and nucleic acids in a membrane-delimited format that protects cargo, enables cell type-specific delivery, and supports multi-node signaling [29,30,31]. Foundational work established EV roles in immune modulation and intercellular transfer of genetic information [32,33,34]. Consensus standards (MISEV 2018; a position statement of the International Society for Extracellular Vesicles and update of the MISEV2014 guidelines. International Society for Extracellular Vesicles: Mount Royal, NJ, USA, 2018, 08061. /MISEV 2023; from basic to advanced approaches. International Society for Extracellular Vesicles: Mount Royal, NJ, USA, 2024, 08061.) and improved fractionation workflows have clarified EV heterogeneity and key confounders, which are particularly important in complex biofluids [35,36,37,38]. In cancer, tumor-derived EVs can modulate immunity, precondition metastatic niches, and determine organotropic phenotypes, providing a mechanistic template for long-range systemic effects [39,40,41,42]. In parallel, the gut microbiota releases bacterial extracellular vesicles—including Outer Membrane Vesicles (OMVs)—that can carry lipopolysaccharide (LPS) and virulence-associated cargo [43,44]. Across diverse microbes, vesicle-mediated delivery contributes to virulence and immune modulation, providing plausible routes for host innate immune engagement even in the absence of live bacteria [45,46,47,48]. In CRC, where microbial ecology, mucosal immunity, and systemic inflammation are closely intertwined, EV-mediated communication provides a mechanistic substrate for cross-kingdom signaling that may contribute to the initiation, progression, and heterogeneity of cachexia [49,50].

**Figure 1:**
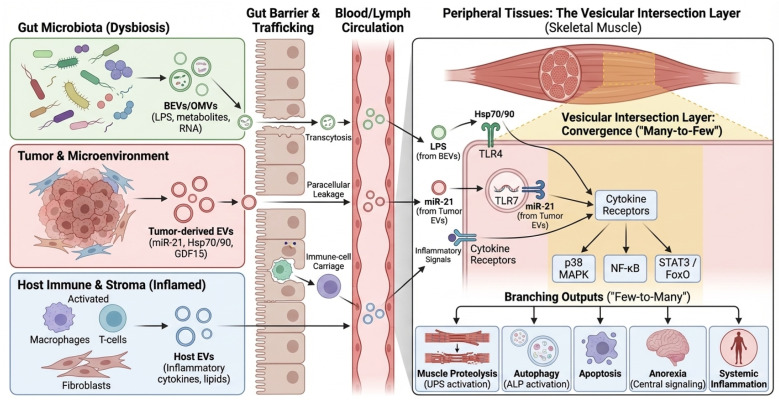
Conceptual overview of the vesicular ecosystem in CRC cachexia. Diagram illustrating the systemic ecosystem of extracellular vesicles (EVs) in colorectal cancer (CRC) cachexia. Colored boxes and vesicles represent distinct source domains: green for gut microbiota (dysbiosis), red for tumor and its microenvironment, and blue for host immune and stromal cells. Black solid arrows indicate the direction of EV trafficking from primary sources through the circulation to peripheral skeletal muscle. In the peripheral tissue (right panel), the yellow-shaded ‘Vesicular Intersection Layer’ denotes the convergence (“Many-to-Few”) of heterogeneous signals onto shared host decoding hubs (e.g., TLR4, TLR7). Branching black arrows at the bottom illustrate the “Few-to-Many” outputs leading to muscle wasting, anorexia, and systemic inflammation. BEV, Bacterial Extracellular Vesicle; CRC, Colorectal Cancer; EV, Extracellular Vesicle; FoxO, Forkhead box O; GDF15, Growth Differentiation Factor 15; Hsp, Heat Shock Protein (potentially EV-associated; may reflect co-isolation); LPS, Lipopolysaccharide; MAPK, Mitogen-Activated Protein Kinase; miRNA, microRNA; NF-κB, Nuclear Factor kappa-light-chain-enhancer of activated B cells; OMV, Outer Membrane Vesicle; STAT3, Signal Transducer and Activator of Transcription 3; TLR, Toll-like Receptor.

In this review we (i) summarize current evidence supporting cross-kingdom EV signaling in CRC cachexia, (ii) introduce the “vesicular intersection layer” as a unifying framework in which diverse EV cargos converge on shared decoding hubs to trigger muscle catabolism, (iii) outline minimum evidentiary standards for causal claims, and (iv) translate the framework into biomarker and therapeutic opportunities. Our focus is deliberately mechanism-to-translation: we aim to help the field move from plausible association to testable, clinically actionable models. Where discussed, endocrine mediators such as GDF15 are treated as predominantly soluble signals, with any vesicular association considered fractional and mechanistically underexplored. A conceptual overview of the proposed vesicular ecosystem and the ‘vesicular intersection layer’ is shown in Figure 1.

This article is a narrative, mechanism- and methodology-focused review; it does not follow a formal systematic review protocol. However, to minimize selection bias, we prioritized literature published between January 2010 and December 2024 indexed in PubMed and Web of Science. Search terms included combinations of “Extracellular Vesicles,” “Exosomes,” “OMVs,” “Colorectal Cancer,” “Cachexia,” “Sarcopenia,” “Gut Microbiota,” and “TLR4.” We specifically prioritized studies that included mechanistic validation (in vitro or in vivo) over purely descriptive association studies, although key clinical correlation studies were included to contextualize translational relevance.

## 2. Definitions, Taxonomy, and Minimum Evidentiary Standards for Cross-Kingdom EV Claims

EV nomenclature and attribution are not semantic details; they determine whether a study supports causal inference or only association. We adopt MISEV2023 as the baseline for methodological reporting and interpretation [37]. We use operational definitions (e.g., ‘small EVs’ defined by size and separation method) rather than assuming biogenesis-based terms, such as ‘exosome’, unless specific evidence is provided [36,37,51]. For CRC cachexia, attribution is further complicated because key biofluids (stool, plasma) contain abundant non-vesicular particles that co-isolate with EVs, particularly lipoproteins and protein aggregates [35,38]. Rigorous fractionation and compositional studies demonstrate that some RNA and protein signals previously attributed to exosomes can originate from non-vesicular carriers, such as HDL, underscoring the need for orthogonal separation strategies [52,53,54]. Furthermore, in applying these standards, we critically evaluate causal direction. Many extant studies rely on exposure-response associations (e.g., adding EVs induces atrophy). However, true causality requires ‘necessary and sufficient’ evidence, such as the ablation of the effect upon blocking EV release (e.g., via GW4869 or genetic Rab knockouts) or specific receptor blockade. In our review, we distinguish between studies that demonstrate this causal loop and those that show only phenotypic association. Comparative features and common analytical pitfalls for host EVs and microbiota-derived vesicles are summarized in Table 1.

We recommend treating cross-kingdom EV statements as hypothesis statements unless they satisfy a minimum set of evidentiary criteria (Box 1). At a minimum, studies should report key pre-analytical variables (collection, storage, freeze–thaw cycles, diet, and antibiotic exposure) and adhere to established EV reporting guidelines [36,37,51]. Investigators should also demonstrate particle enrichment and purity using orthogonal assays (e.g., electron microscopy, protein-to-particle ratios, and negative markers) rather than relying on a single isolation method [91,95]. Because stool and plasma contain abundant non-vesicular particles, major confounders—particularly lipoproteins and endotoxin/LPS carryover—should be explicitly assessed for preparations used in functional assays [35,38]. Where mechanistic claims depend on topology, cargo localization (membrane vs. lumen) should be demonstrated rather than assumed [57]. Finally, causal direction should be established using perturbation approaches (such as genetic or pharmacological inhibition of EV biogenesis, release, or uptake) instead of correlation alone [36,37,51,96].

Box 1 **Minimum evidentiary standards for cross-kingdom EV mechanisms in CRC cachexia.**

(1) Attribution: demonstrate whether the functional preparation is host-derived EVs, BEVs/OMVs, or mixed (e.g., 16S/shotgun metagenomics on vesicle-associated nucleic acids; bacterial vs. host membrane markers; density/charge-based separations).
(2) Purity: quantify and report major co-isolates (lipoproteins, protein aggregates). When plasma is used, explicitly address HDL/LDL co-isolation [35,38,53].
(3) Cargo localization: use RNase/protease protection assays with and without detergents to distinguish surface-adsorbed vs. luminal cargo when RNA or proteins are proposed as effectors [36,37,51].
(4) Functional specificity: control for endotoxin or LPS carryover in vitro (e.g., polymyxin B controls are insufficient alone); compare dose–response against matched particle counts.
(5) Causality: perturb EV release/uptake (e.g., genetic inhibition of secretion machinery; receptor blockade; uptake inhibitors) and link to cachexia-relevant endpoints in vivo (muscle mass, function, and catabolic gene expression).
(6) Clinical anchoring: in humans, prioritize longitudinal sampling and outcomes aligned with consensus cachexia definitions [4,5,6,7]. Ensure phenotyping aligns with significant clinical practice guideline recommendations [10,11]. Utilize endpoint frameworks that integrate imaging-based muscle measures with functional readouts to enhance trial interpretability [94]. Imaging-derived body composition metrics (including CT-based muscle indices) are prognostic and provide objective longitudinal endpoints [16,17,18,19,20]. Because chemotherapy can directly contribute to muscle loss and systemic stress, treatment exposures should be recorded and incorporated into analyses [82,83,88]. Microbiome-relevant covariates (dietary patterns and antibiotic use) should also be reported to support cross-cohort comparability [25,26,27,97]. Supportive-care interventions that modify appetite and metabolism, including pharmacologic approaches and structured exercise, should be captured alongside EV measurements [98,99,100,101].

To illustrate how these minimum standards change interpretation, we provide a qualitative appraisal of representative studies in CRC cachexia and closely related models using the Box 1 criteria (Supplementary Table S1). This is not a systematic review, but a transparency tool to highlight where attribution, purity controls, topology, and in vivo cachexia anchoring are strong versus where key gaps remain.

## 3. The CRC Cachexia Vesicular Ecosystem: Defining the ‘Vesicular Load’ Across Tumor and Microbial Domains

A vesicular ecosystem perspective emphasizes that biologically relevant exposure encompasses not only microbial abundance but also vesicle production, stability, and trafficking—collectively, the ‘vesicular load’. We propose ‘vesicular load’ as a hypothetical composite metric anchored to (i) BEV/OMV particle abundance and (ii) vesicle-associated microbial signatures (e.g., vesicle-associated 16S/shotgun), interpreted alongside barrier status and key clinical covariates. It is important to note that validated assays for quantifying systemic exposure to gut-derived vesicles in humans have not yet been established, and this concept requires standardization before clinical application. This perspective is consistent with CRC microbiome studies and meta-analyses that report reproducible, cohort-level shifts in microbial signatures and functional potential [25,26,27,97]. Two patients with comparable fecal abundance of a given microbe may nevertheless have different systemic exposure to that microbe’s vesicles if epithelial permeability, mucosal inflammation, or host clearance differs [74,75,76].

Key EV-producing compartments in CRC cachexia include tumor tissue and the circulation, host immune and stromal compartments, metabolic organs (such as the liver and adipose tissue), and the gut microbiota. Tumor-derived EVs can modulate immunity and shape distal tissue responses, supporting a mechanism for systemic phenotypes [39,40,41,42]. CRC-associated dysbiosis is reproducible across cohorts and is linked to inflammation and tumor-promoting signaling [23,24]. As a well-studied exemplar among multiple CRC-associated pathobionts, *F. nucleatum* is repeatedly enriched in CRC tissues and stool across independent studies [59,61,69]. Mechanistically, *F. nucleatum* can promote oncogenic signaling and immune evasion, and it has been linked to chemoresistance and altered tumor immunity [60,62,66,71,72]. Importantly for cross-kingdom signaling, *F. nucleatum* produces OMVs that can promote intestinal inflammation, providing a vesicular route for microbial influence beyond direct bacterial invasion [64].

Importantly, many cachexia phenotypes—such as systemic inflammation and metabolic dysregulation—are processes in which EV signaling has established roles in other disease settings [52,53,54,58]. Cancer-associated EVs can remodel distant tissues and immune compartments, supporting the plausibility of EV-mediated propagation of systemic phenotypes [39,40,41,42]. In parallel, microbial PAMP signaling (including metabolic endotoxemia and TLR pathway activation) provides a mechanistic bridge between gut ecology and host metabolism [77,78,80,81]. Thus, CRC cachexia is a setting in which EV biology and gut ecology are likely to reinforce one another.

## 4. From Lumen to Muscle: EV Trafficking Routes and Barrier Gating

**Figure 2:**
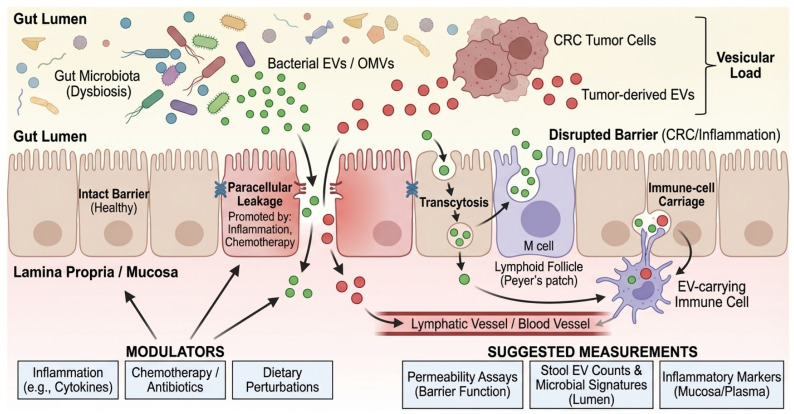
Barrier gating and cross-kingdom EV trafficking routes. Schematic of intestinal epithelial barrier routes and their modulators in CRC patients. Green vesicles represent bacterial EVs/OMVs originating from the gut lumen, while red vesicles represent tumor-derived EVs. Black arrows indicate the three primary trafficking routes across the intestinal epithelium: transcytosis (via enterocytes or M cells), paracellular leakage (facilitated by disrupted tight junctions), and immune-cell carriage. Blue ‘X’ symbols on tight junctions denote barrier disruption promoted by inflammation or chemotherapy. The gradient from the gut lumen to the blood vessel illustrates the systemic translocation of the ‘Vesicular Load’. BEV, Bacterial Extracellular Vesicle; CRC, Colorectal Cancer; EV, Extracellular Vesicle; LPS, Lipopolysaccharide; M cell, Microfold cell; OMV, Outer Membrane Vesicle; PBMC, Peripheral Blood Mononuclear Cell.

Cross-kingdom EV signaling requires a physical route from the gut lumen to systemic compartments. Proposed routes include (i) transcytosis across epithelial cells, including M cells over Peyer’s patches; (ii) paracellular leakage under tight junction disruption; and (iii) uptake by mucosal immune cells with subsequent lymphatic or hematogenous dissemination [74,75,76]. Beyond these general routes, mechanisms of cellular recognition differ by vesicle origin. Gram-negative OMVs are frequently internalized by non-phagocytic cells via clathrin-mediated endocytosis and lipid raft-dependent fusion, processes often triggered by the interaction of vesicular LPS with host TLR4 [47]. Conversely, Gram-positive MVs can deliver cargo via membrane fusion or interaction with TLR2. In the context of muscle, recent studies suggest that EVs may also exploit the ‘gut-lymph’ axis, bypassing first-pass hepatic clearance to reach systemic circulation and peripheral tissues more effectively than soluble mediators. CRC-related inflammation, chemotherapy, antibiotic exposure, and dietary perturbations can all modulate these routes, complicating attribution in both human cohorts and animal models. Furthermore, a major quantitative feasibility gap remains: even if barrier disruption facilitates translocation, it is currently unknown whether sufficient quantities of intact bacterial EVs bypass hepatic clearance and dilution in the systemic circulation to reach skeletal muscle and trigger catabolism. Most mechanistic evidence relies on high-dose administration in mice; thus, physiological relevance in humans awaits sensitive biodistribution studies. A schematic of barrier gating and proposed EV trafficking routes from the lumen to the muscle is shown in Figure 2. To facilitate implementation in human CRC cachexia cohorts, we summarize a prioritized minimal measurement set spanning stool and plasma EV/BEV readouts, barrier context, and longitudinal cachexia phenotyping (Box 2). However, it is crucial to acknowledge that although trafficking routes (transcytosis, paracellular leak) are well established in colitis and sepsis models, direct evidence that CRC-associated microbial vesicles physically reach and degrade skeletal muscle tissue remains a key knowledge gap that requires validation in specific CRC mouse models.

Box 2 **Suggested minimal measurement set for human CRC cachexia cohorts (prioritized).**

- **Tier 1 (core; recommended for most studies):** (i) Standardized stool EV/BEV enrichment with particle counts (normalized to input mass/volume) and process blanks; (ii) Vesicle-associated microbial signatures (16S/shotgun on vesicle-associated nucleic acids) with negative controls; (iii) Plasma EV preparations with explicit assessment of major co-isolates (especially lipoproteins) and endotoxin/LPS carryover when used for functional inference; (iv) Longitudinal cachexia phenotyping anchored to consensus definitions (CT-derived muscle indices ± strength/function) and aligned sampling timepoints; (v) Key metadata capturing chemotherapy, antibiotics, diet, and supportive-care interventions.
- **Tier 2 (enhanced mechanistic anchoring):** (i) Barrier/permeability readouts to contextualize translocation (“barrier gating”); (ii) Inflammatory panels and PBMC signatures consistent with PRR–p38/NF-κB activation; (iii) Orthogonal EV characterization for functional preparations (e.g., EM and protein-to-particle ratios) and negative-marker reporting.

Beyond physical permeability, trafficking across the gut–blood interface is constrained by immunological “gating” at pattern-recognition receptors (PRRs), with TLR4 and endosomal TLR7/8 functioning as candidate decoding hubs for vesicle-associated microbial and host-derived ligands that converge on shared catabolic programs in skeletal muscle [77,80,81]. EV-associated RNAs, including microRNAs, can directly engage endosomal TLRs and amplify inflammatory responses [65]. In parallel, LPS-driven TLR4 signaling can directly promote skeletal muscle catabolism through the coordinated activation of the ubiquitin–proteasome system and autophagy–lysosomal pathways [79]. This links gut-derived inflammatory signals to the core muscle-wasting machinery defined in classical cachexia models [15,102,103,104,105].

## 5. The Vesicular Intersection Layer: Convergence of EV Cargo on Shared Decoding Hubs

We define the vesicular intersection layer as the set of molecular interfaces where heterogeneous EV cargos—including tumor-derived factors, host inflammatory mediators, and microbial vesicle–associated ligands—converge on shared host decoding hubs to activate a common set of downstream catabolic programs in skeletal muscle. In simpler terms, the ‘intersection layer’ functions like a funnel: diverse stress signals from the tumor and the gut microbiome are packaged into vesicles, travel through the circulation, and ultimately trigger the same ‘alarm’ switches (receptors) on muscle cells. Conceptually, it follows a many-to-few-to-many architecture: diverse upstream vesicle sources feed into a limited set of receptors and signaling nodes, which then branch into multiple muscle and systemic outputs (e.g., proteolysis, autophagy, inflammatory amplification, and metabolic dysregulation). What this framework adds is an explicit carrier dimension—EV provenance, topology, co-isolates, and trafficking constraints—thereby linking mechanistic hypotheses to actionable measurement strategies (such as vesicular load and vesicle-associated microbial signatures) and to minimum evidentiary standards required for cross-kingdom attribution (Box 1).

Among these shared decoding hubs that define the intersection layer, TLR4 and endosomal RNA-sensing TLRs emerge as the most consistently supported candidates in CRC cachexia–relevant studies. While canonical models focus on LPS-TLR4 signaling, the vesicular intersection layer extends beyond Gram-negative bacteria. Membrane vesicles (MVs) released by Gram-positive pathobionts (e.g., *Enterococcus* spp.), which are increasingly implicated in CRC dysbiosis, transport lipoteichoic acid (LTA) and other ligands that can engage TLR2. Mechanistically, this reinforces the concept of the intersection layer, as TLR2—like TLR4—recruits MyD88 to activate downstream NF-κB and p38 MAPK pathways. This ensures that diverse vesicular cargos, whether Gram-positive (LTA) or Gram-negative (LPS), trigger common muscle catabolic programs. This suggests a broader ‘many-to-few’ convergence where various PRRs (TLR2, TLR4, TLR7/8) integrate signals from the diverse CRC microbiome. Several tumor- and host-derived EV cargos can engage these axes. For example, microvesicle-associated miR-21 can activate TLR7 signaling to promote myoblast apoptosis and muscle wasting in vivo, providing a direct link between EV cargo and muscle catabolism [84]. Extracellular Hsp70 and Hsp90 released in EV-associated or extracellular forms can activate TLR4 and drive p38 MAPK-dependent muscle wasting in general cancer cachexia models [86]. Microbial vesicle cargos, particularly LPS displayed on Gram-negative OMVs, are canonical TLR4 ligands and can provide a high-avidity PAMP stimulus when delivered in vesicular form [43,44,46,47]. In the host, TLR4 integrates microbial PAMPs with sterile inflammatory cues and has been implicated in metabolic endotoxemia and innate immune priming [77,78,80,81]. It is important to distinguish between proven and hypothetical pathways in this context: while BEV-driven systemic inflammation (indirect action) is a well-established driver of muscle catabolism via cytokine release, the physical translocation of intact BEVs to the sarcolemma for direct TLR4 engagement (direct action) remains a plausible but yet-to-be-proven hypothesis in human CRC patients. Notably, TLR4 activation—whether by direct vesicular contact or systemic inflammatory mediators—is sufficient to drive muscle catabolic programs, and pharmacologic blockade with resatorvid (TAK-242) has shown preclinical efficacy in attenuating cancer-associated muscle atrophy [79,89,90].

**Figure 3:**
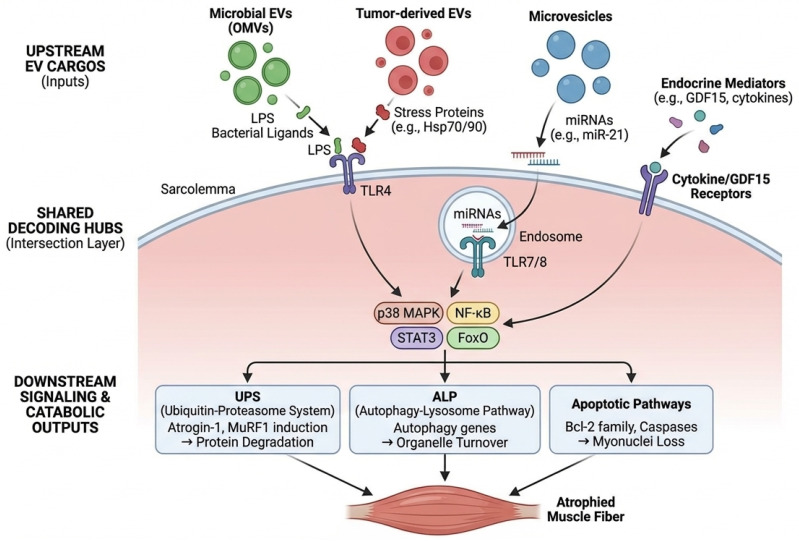
The vesicular intersection layer in skeletal muscle. Molecular map of candidate EV cargos converging on shared decoding hubs within the skeletal muscle fiber. The light-blue arc represents the sarcolemma (muscle cell membrane), and the pink-shaded interior represents the sarcoplasm. Pointed black arrows (→) signify activation of signaling pathways or induction of gene expression. Specific cargos are color-coded by origin: green for microbial LPS/ligands, red for tumor-derived stress proteins, and blue for microvesicle-associated miRNAs. The diagram highlights the convergence of these diverse inputs onto shared signaling nodes (p38 MAPK, NF-κB, STAT3, FoxO), which subsequently drive the primary catabolic effectors of muscle atrophy. ALP, Autophagy-Lysosome Pathway; Bcl-2, B-cell lymphoma 2; BEV, Bacterial Extracellular Vesicle; FoxO, Forkhead box O; GDF15, Growth Differentiation Factor 15; LPS, Lipopolysaccharide; MAPK, Mitogen-Activated Protein Kinase; miRNA, microRNA; NF-κB, Nuclear Factor kappa-light-chain-enhancer of activated B cells; OMV, Outer Membrane Vesicle; STAT3, Signal Transducer and Activator of Transcription 3; TLR, Toll-like Receptor; UPS, Ubiquitin-Proteasome System.

Downstream, canonical cachexia pathways—NF-κB, p38 MAPK, STAT3/JAK, and FoxO—serve as convergence points for diverse upstream inputs [102,103,104,105]. In muscle, these nodes induce the ubiquitin–proteasome system and autophagy–lysosome programs through E3 ligases (MuRF1, Atrogin-1) and autophagy regulators, and they can also engage pro-apoptotic signaling [102,103,105]. Clinical and preclinical evidence indicates that both cancer and its treatments, including chemotherapy, can further amplify these catabolic programs and confound biomarker readouts [82,83,88]. This creates a plausible ‘intersection layer’ in which vesicular inputs from multiple compartments converge on a limited set of catabolic executors, even if individual cargo species differ across patients (Section 8). A schematic summary of shared decoding hubs and downstream catabolic execution programs is provided in Figure 3.

## 6. CRC-Relevant Vesicular Mediators: What Is Established, What Is Plausible, and What Is Unproven

Key candidate vesicular mediators, their decoding hubs, and translational touchpoints are summarized in Table 2.

### 6.1. Microbiota-Derived Vesicles and CRC-Associated Dysbiosis

CRC-associated dysbiosis is reproducible across cohorts, with consistent enrichment of microbial taxa and functions linked to inflammation and tumor-promoting signaling [23,24]. These consistent shifts in the microbiome imply a parallel alteration in the luminal vesicular landscape, potentially creating a distinct “EV signature” in CRC patients [25,26,27]. Conceptual frameworks, such as the bacterial driver–passenger model, further highlight how microbial ecology can shift during CRC progression [75]. As a well-studied exemplar rather than an exclusive driver, *F. nucleatum* is repeatedly enriched in CRC tissues and stool across independent studies [59,61,69]. Importantly, it produces OMVs that can promote intestinal inflammation, providing a vesicular route for microbial influence beyond direct bacterial invasion [64]. However, CRC dysbiosis is not limited to Gram-negative anaerobes. Gram-positive pathobionts, such as Enterococcus faecalis, are also enriched in the CRC tumor microenvironment and produce extracellular membrane vesicles (MVs). Unlike Gram-negative OMVs, these MVs lack LPS but are enriched in lipoteichoic acid (LTA) and hemolysin/cytolysin, which are potent agonists of TLR2 and NLRP3 inflammasomes. This highlights that the ‘vesicular load’ in CRC likely comprises a mixture of TLR4-activating (LPS) and TLR2-activating (LTA) signals, both of which converge on the NF-κB catabolic node in skeletal muscle. Notably, the ColoCare study reports an association between *F. nucleatum* abundance and cachexia-related phenotypes [67]; however, this observation should be treated as hypothesis-generating given potential confounding by disease stage, treatment exposures, dietary shifts, and systemic inflammation, underscoring the need for longitudinal designs and mechanistic perturbations (Section 9). Beyond LPS-rich membranes, BEVs/OMVs can also carry regulatory RNAs, including tRNA-derived fragments and other small RNAs, which can reprogram host immune pathways in experimental systems, expanding the space of candidate cross-kingdom effectors [63,68], reinforcing the value of integrative concepts (e.g., vesicular load and shared decoding hubs) over attributing cachexia risk to a single microbe.

### 6.2. Tumor-Derived and Host EVs as Direct Effectors of Muscle Catabolism

Multiple studies support the concept that tumor-derived EVs can directly impair muscle homeostasis. Microvesicles containing miR-21 were shown to activate TLR7 signaling and induce myoblast apoptosis, promoting muscle wasting in experimental cachexia [84]. While this provides a direct link between EV cargo and muscle catabolism [80], it is important to acknowledge that RNA-mediated TLR activation by EVs has faced reproducibility challenges in the broader field. Whether vesicular miRNA concentrations in human plasma reach the threshold required for TLR7 activation remains to be fully established. Separately, extracellular Hsp70/Hsp90, which may be vesicle-associated or co-isolated with EVs, has been shown to activate TLR4–p38 pathways to drive muscle catabolism [86], aligning with broader data connecting TLR4 activation to proteasome and autophagy pathways in muscle [79].

Beyond inflammatory decoding hubs, endocrine mediators packaged or associated with EVs may amplify systemic cachexia. While GDF15 is predominantly a soluble cytokine, a fraction of circulating GDF15 may be vesicle-associated or co-transported. Tumor-derived exosomal GDF15 has been implicated in muscle atrophy in a colon cancer cachexia model [87]. However, given that GDF15 is primarily a soluble cytokine, careful separation is required to determine whether the vesicle-associated fraction represents specific encapsulation or non-specific adherence, as noted in recent methodological critiques [35,38]. Independent of vesicular association, GDF15 signals through the brainstem receptor GFRAL to suppress appetite and reduce body weight, establishing a biologically validated anorexia axis [106,107,108,109]. The clinical relevance of this axis was underscored by a phase 2 randomized trial of the anti-GDF15 antibody ponsegromab, which increased body weight and improved patient-reported outcomes in cancer cachexia, with CRC among represented tumor types [110].

These findings motivate a coherent hypothesis: tumor EVs and BEVs/OMVs may jointly elevate the probability and intensity of cachexia by (i) converging on shared inflammatory hubs (TLR4, p38, NF-κB) and (ii) coupling peripheral catabolism with central anorexia signals (GDF15–GFRAL). Testing this requires integrated measurement of EV cargo, microbial signatures, barrier status, and clinical cachexia endpoints.

## 7. EV-Informed Endotyping and Biomarker Strategy

Throughout this review, we distinguish (i) stool/plasma EV signatures associated with CRC detection or prognosis from (ii) EV measures linked to cachexia endpoints (e.g., longitudinal CT-derived muscle indices, strength and function, and/or activation of catabolic programs). Because most human EV biomarker studies in CRC were not designed around cachexia phenotyping, CRC association alone should not be interpreted as cachexia causality. Accordingly, we explicitly annotate evidence levels in Table 2 and prioritize longitudinal designs anchored to consensus cachexia definitions and clinical phenotyping standards (Box 1). Cachexia is biologically heterogeneous, with substantial inter-patient variability in dominant drivers and therapeutic responsiveness, motivating endotype-aware biomarker strategies. Practically, ‘endotyping’ refers to classifying patients into subgroups based on the specific biological mechanism driving their wasting (e.g., bacterial inflammation vs. tumor-derived metabolic stress), rather than just their clinical symptoms, enabling more targeted treatments. Crucially, these EV-defined endotypes are not distinct from, but rather upstream drivers of, the classical cellular mechanisms of muscle wasting. For instance, the ‘Inflammatory Endotype’ (high microbial vesicular load) is hypothesized to drive muscle wasting primarily through TLR4-mediated production of Reactive Oxygen Species (ROS) and subsequent mitochondrial dysfunction. Conversely, the ‘Anorexia Endotype’ induces a state of negative energy balance, which indirectly impairs mitochondrial quality control (mitophagy). Therefore, EV endotyping identifies the specific ‘trigger’ in a given patient, while the downstream consequences—oxidative stress and mitochondrial failure—remain shared.

**Figure 4:**
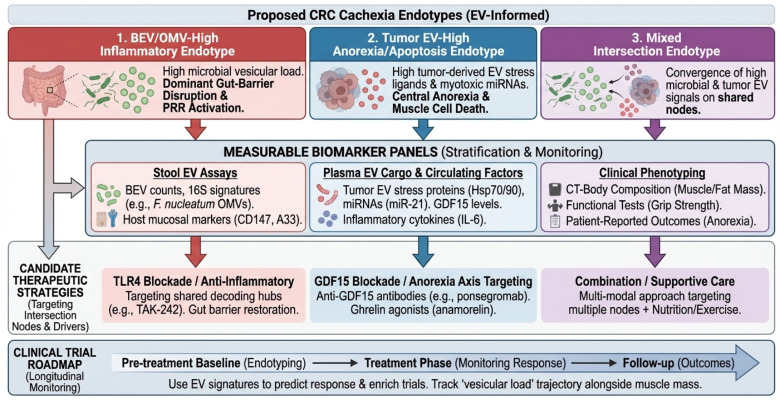
Proposed endotypes and a roadmap for biomarker-driven trials. Clinical translation framework mapping EV-informed endotypes onto measurable biomarker panels and therapeutic strategies. The three top boxes define distinct patient endotypes, color-coded for clinical stratification: red for BEV/OMV-high inflammatory, blue for tumor EV-high anorexia/apoptosis, and purple for the mixed intersection endotype. Vertical colored arrows indicate the flow from patient endotyping to specific biomarker panels (stool, plasma, and clinical phenotyping). The ‘Clinical Trial Roadmap’ at the bottom (large blue arrow) illustrates the longitudinal monitoring schema from pre-treatment baseline through follow-up.A33, GPA33 (Cell surface glycoprotein A33); BEV, Bacterial Extracellular Vesicle; CD147, Basigin (Cluster of Differentiation 147); CRC, Colorectal Cancer; CT, Computed Tomography; EV, Extracellular Vesicle; GDF15, Growth Differentiation Factor 15; IL-6, Interleukin 6; LPS, Lipopolysaccharide; OMV, Outer Membrane Vesicle; PRR, Pattern Recognition Receptor; SMI, Skeletal Muscle Index; TLR, Toll-like Receptor.

We propose three pragmatic endotypes as a starting point (to be refined empirically): (i) BEV/OMV-high inflammatory endotype (high microbial vesicular load and PRR activation), (ii) tumor EV-high anorexia/apoptosis endotype (enrichment for tumor EV-associated stress ligands and microRNAs with myotoxic effects), and (iii) mixed intersection endotype (both high microbial vesicles and tumor EV signals). Technically, differentiating this ‘mixed’ endotype from single-source phenotypes is achievable through multiplexed liquid biopsy assays. For example, combining digital PCR for bacterial 16S rRNA genes with specific tumor-derived miRNA signatures (e.g., methylated DNA markers or exosomal miR-21) within the same isolated EV fraction can resolve the dual contribution of the microbiome and the tumor. However, consistent with Box 1, detection of both signals in a bulk isolate does not prove they reside within the same vesicle. Rather, it indicates the concurrent presence of distinct tumor-derived and microbial vesicle populations (or co-isolates) in the circulation, which collectively define the patient’s ‘mixed’ inflammatory and metabolic risk profile. These are explicitly testable constructs that can be mapped onto measurable markers and outcomes. EV-informed endotypes and a roadmap for biomarker-driven trials are summarized in Figure 4.

Stool is an underused biospecimen for cachexia biology. In CRC, fecal EVs have been explored as diagnostic and prognostic biomarkers; for example, a study identified fecal EV markers (including CD147 and A33) with performance exceeding traditional serum markers in specific settings [93]. Large-scale profiling suggests that stool-derived EVs can provide a proteomic readout of the gut environment, creating opportunities for ‘vesiculomics’ panels [92]. However, stool presents a harsh biochemical environment. The presence of bile salts, bacterial proteases, and nucleases can rapidly degrade EV membranes and cargo. Therefore, robust protocols including immediate stabilization (e.g., protease inhibitor cocktails) and rigorous quality control to distinguish intact vesicles from bacterial debris are essential for valid fecal EV analysis. Future studies should investigate whether stool EV signatures predict the trajectory of pre-cachexia and cachexia, and whether they provide additional value beyond metagenomic abundance alone (Section 3).

For plasma biomarker strategies, it is essential to acknowledge that EV preparations can be confounded by lipoproteins and other nanoparticles, necessitating standardized separation and reporting [35,38,53,91]. Integrated panels that combine EV-associated markers (e.g., GDF15 axis signals, stress proteins, and vesicle-associated microbial signatures) with imaging-based muscle phenotyping and systemic inflammatory markers are more likely to stratify biologically heterogeneous patients than any single analyte [6,94]. Such stratification should be anchored to consensus cachexia definitions [4,5,6,7]. Guideline-based phenotyping further improves clinical interpretability and reproducibility [10,11]. In CRC, CT-based body composition measures can be extracted from routine imaging and provide objective endpoints for longitudinal validation [16,17,18,19,20].

## 8. Therapeutic Opportunities: Targeting Intersection Nodes and Reshaping the Vesicular Ecosystem

### 8.1. Targeting Shared Decoding Hubs

The intersection-layer framework emphasizes shared, targetable nodes. TLR4 is a high-priority candidate because it integrates microbial PAMPs with sterile stress cues, providing a mechanistically defined entry point into innate immune signaling [77,78,80,81]. In skeletal muscle, TLR4 activation can coordinate ubiquitin–proteasome and autophagy programs, and tumor-associated extracellular Hsp70/Hsp90 can serve as endogenous ligands that reinforce this axis [79,86]. TAK-242 (resatorvid) is a small-molecule TLR4 signaling inhibitor that binds to TLR4 and disrupts adaptor interactions [89]. Preclinical data suggest that TAK-242 can attenuate cancer-associated muscle atrophy via p38–C/EBPβ signaling, supporting the concept that blocking an intersection node can reduce muscle wasting even in complex systemic contexts [90]. Nevertheless, translational caution is warranted. Previous trials targeting single cytokines (e.g., TNF-α) in cancer cachexia have largely failed, likely due to the pathway redundancy inherent in the ‘many-to-few’ model described here. Furthermore, systemic inhibition of central nodes, such as TLR4, carries significant risks of immunosuppression, which could impair anti-tumor immunity. Therefore, the therapeutic value of this framework may lie not in broad monotherapy but in using EV profiles to stratify patients who are specifically driving wasting via these inflammatory hubs, as opposed to those driven by endocrine or metabolic defects.

### 8.2. Targeting Endocrine and Central Appetite Axes

GDF15–GFRAL signaling is a validated anorexia axis with direct clinical translation. While GDF15 functions primarily as a soluble cytokine, its potential association with EVs suggests a mode of delivery that may protect the ligand or enhance its bioavailability. However, within the ‘intersection layer’ framework, the critical point is not solely the transport vehicle, but the functional integration: GDF15-mediated anorexia signals converge with EV-driven inflammatory catabolism (e.g., TLR4 activation) to amplify the wasting phenotype. Thus, while the vesicular association of GDF15 remains mechanistically underexplored, the axis itself is a tractable therapeutic target, as shown by ponsegromab in cancer cachexia [106,107,108,109,110]. From an EV perspective, the key translational question is whether EV-associated measurements improve the prediction of response or identify subgroups whose cachexia is dominated by anorexia rather than peripheral inflammatory catabolism.

### 8.3. Modulating EV Release, Uptake, and the Microbiota

Broadly inhibiting EV biogenesis or uptake is conceptually attractive but clinically challenging due to pleiotropic roles of EVs in homeostasis and immunity. Nevertheless, experimental inhibition of tumor EV-related stress pathways (e.g., reducing extracellular Hsp70/Hsp90 release) has been proposed as a strategy to mitigate cachexia [111]. At the ecosystem level, exposures that remodel the gut microbiome—such as dietary patterns and antibiotic use—are expected to alter microbial vesicular load and barrier-related translocation dynamics [23,24,25,26,27]. In CRC patients receiving chemotherapy, such strategies must be evaluated for safety and interaction with treatment-related catabolic stress [82,83,88]. In parallel, supportive-care interventions that modify appetite and metabolism (including pharmacologic approaches and structured exercise) should be integrated into cachexia trial design and covariate reporting [98,99,100,101].

Finally, supportive care remains foundational. Ghrelin receptor agonism (anamorelin) has shown benefits for appetite and body weight in phase 3 trials for cancer cachexia, illustrating that multimodal management is likely required even if EV-targeted therapies succeed [98,99,100,101].

## 9. Research and Clinical Roadmap: From Association to Causality

To move the field forward, studies should be designed to answer specific causal questions rather than only describing associations. Priority designs include: (i) longitudinal CRC cohorts with repeated stool and plasma collection before and during treatment, coupled to standardized cachexia phenotyping (body composition, functional measures, inflammatory markers), (ii) mechanistic mouse models combining defined microbiota (including gnotobiotic systems) with tumor implantation and vesicle perturbation, and (iii) ex vivo human systems (organoids, muscle microtissues) for mechanistic dissection with controlled EV exposures. Future studies must rigorously test the ‘direct contact’ hypothesis by utilizing labeled BEVs/MVs to track biodistribution to skeletal muscle, distinct from the effects of systemic cytokines. Furthermore, expanding mechanistic studies to include Gram-positive vesicles and TLR2 signaling will ensure the model captures the full breadth of CRC dysbiosis.

Endpoints should be clinically meaningful and mechanistically linked. CT-based body composition quantification is feasible in CRC because imaging is routinely obtained, enabling precise tracking of muscle and adipose compartments and supporting cachexia staging and trial endpoints [16,17,18,19,20]. Harmonized definitions of cachexia are essential for cross-study comparability and external validation [4,5,6,7]. Guideline-based phenotyping further improves reproducibility across cohorts [10,11]. Endpoint selection for trials should follow established cachexia trial frameworks [94]. EV workflows should follow MISEV reporting and include contaminant controls, which are prerequisites for meta-analyses and biomarker validation [36,37,51]. The current framework’s limitations must also be acknowledged. First, separating host-derived EVs from bacterial OMVs in human stool and plasma remains technically challenging due to overlapping sizes and densities. Second, much of the mechanistic evidence linking OMVs to muscle catabolism is derived from sepsis or sterile inflammation models rather than CRC-specific models. Finally, this review is narrative in nature; systematic reviews of clinical trial data will be necessary once more standardized EV quantification methods are adopted in cachexia cohorts. To translate this research roadmap into specific experimental inquiries, we outline the key testable predictions derived from the vesicular intersection layer framework in Box 3.

Box 3 **Hypothesized predictions derived from the vesicular intersection layer framework.**

- **Prediction 1:** In CRC cohorts, ‘vesicular load’ metrics (e.g., BEV/OMV particle counts and vesicle-associated 16S signatures) are hypothesized to correlate with cachexia severity more strongly than bulk microbial abundance alone, after adjustment for antibiotics and chemotherapy.
- **Prediction 2:** Patients with high BEV/OMV load are expected to show preferential activation of TLR4–p38 transcriptional signatures in muscle and peripheral blood mononuclear cells compared with patients with low BEV/OMV load, independent of tumor stage.
- **Prediction 3:** Pharmacologic interruption of a shared intersection node (e.g., TLR4 blockade) is predicted to attenuate muscle catabolism even when upstream EV cargos differ (tumor-derived vs. microbial), whereas cargo-specific blockade will benefit only subsets.
- **Prediction 4:** Stool EV assays may enable earlier detection of CRC-associated systemic perturbations (including signals consistent with pre-cachexia trajectories) than plasma-only biomarkers, because stool is enriched for microbial vesicles and may provide a higher signal-to-noise ratio for vesicle-associated microbial signatures.
- **Prediction 5:** EV-informed endotypes (Section 7) could predict differential responses to cachexia therapies (e.g., GDF15 blockade vs. anti-inflammatory node blockade) and can be used to enrich clinical trials.

## 10. Conclusions

CRC cachexia should be viewed as an emergent systemic phenotype arising from tumor–host–microbiota interactions. EVs are not the only mediators of this crosstalk, but they are capable of transporting multi-modal information across anatomical barriers and engaging shared decoding hubs. The vesicular intersection layer framework clarifies how heterogeneous EV cargos can converge on a limited set of receptors and signaling nodes to drive common muscle-wasting programs. While soluble mediators can also converge on these nodes, the distinctive value of the EV framework lies in its potential for source attribution: unlike generic cytokines, EV cargos (e.g., vesicle-associated microbial nucleic acids and tumor-derived mutant nucleic acids) can carry information about their origin, enabling testable hypotheses about whether cachexia is driven primarily by the tumor, the microbiome, or both. Together, this offers a hypothesis to account for clinical heterogeneity and suggests rational endotypes.

Near-term opportunities include (i) rigorous characterization of microbial and host EV fractions in stool and plasma using standardized methods, (ii) linking vesicular load to cachexia trajectories in well-phenotyped CRC cohorts, and (iii) testing intersection-node therapies (e.g., TLR4 blockade) in preclinical models with clinically relevant outcomes. With these steps, cross-kingdom EV biology can move from an intriguing concept to a practical lever for diagnosis, stratification, and therapy in CRC cachexia.

## Figures and Tables

**Table 1:** Comparative features of host extracellular vesicles versus microbiota-derived vesicles relevant to CRC cachexia.

Feature	Host EVs (sEV-Enriched and m/lEVs)	BEVs/OMVs (Microbiota-Derived Vesicles)	Key Analytical Notes/Pitfalls
Biogenesis	Exosomes: Endosomal pathway (MVB fusion) Microvesicles: plasma membrane budding Refs: [29,30,31,32,33,34,55,56,57].	Gram (−): Outer membrane blebbing Gram (+): Plasma membrane vesiculation (MVs) Refs: [43,44,45,46,48].	Avoid assuming biogenesis solely from size. Use operational terms (e.g., “small EVs”). Refs: [36,37,51].
Typical Size Range	sEVs often 30–150 nm m/lEVs (100–1000 nm) Refs: [36,37,51].	OMVs: Typically 20–250 nm MV: Wider range (species-dependent) Refs: [43,44,45,48].	Substantial overlap exists. Size alone cannot attribute origin.
Membrane Composition	Host lipids (cholesterol, sphingomyelin) Tetraspanins, cell-specific proteins Refs: [30,31,55,56].	Gram (−): LPS, outer membrane proteins Gram (+): LTA, Peptidoglycan Refs: [43,44,45,46,47,48].	Endotoxin/LPS quantification is essential for functional assays. Refs: [35,38]
Canonical Markers	Pos: CD9, CD63, CD81, ALIX, TSG101 Neg: Calnexon, Apolipoproteins Refs: [36,37,51].	Gram (−): OmpA, LPS Gram (+): LTA, surface proteins (no universal BEV marker) Refs: [43,44,45,46,47,48].	Must assess negative markers (e.g., ApoA1/B) for lipoprotein contamination. Refs: [35,38,53].
Dominant Cargo	Proteins, miRNAs, lncRNAs, lipids, metabolites Reflects cell state Refs: [29,30,31,33,34,55,56,58].	LPS, peptidoglycan, bacterial RNAs(sRNA, tRNA fragment), metabolites Virulence factors Refs: [43,44,45,46,47,48,59,60,61,62,63,64,65,66,67,68,69,70,71,72].	Use RNase/protease protection assays to distinguish surface vs. luminal cargo. Refs: [36,37,51].
Trafficking Routes	Local & systemic via blood/lymph Depends on integrins/glycans Refs: [39,40,41,73].	Transcytosis, paracellular leakage, immune-cell carriage Refs: [74,75,76].	Barrier status is a major confounder Hepatic firewall clears most systemic BEVs.
Decoding Hubs	PRRs (TLRs), cytokine receptors Stress-response signaling Refs: [42,65,77,78,79,80,81].	TLR4 (LPS), TLR2 (LTA/peptidoglycan) Other PRRs Refs: [77,78,79,80,81].	Convergence on shared nodes supports intersection-layer interventions.
Relevance to Muscle	Apoptosis, proteolysis, inflammation Metabolic dysfunction Refs: [82,83,84,85,86,87,88].	Systemic inflammation TLR4-dependent catabolic programs Refs: [77,78,79,80,81,89,90].	Direct BEV-to-muscle causality in human CRC remains a key gap. Ref: [49].
Clinical Sampling	Plasma/serum Affected by preanalytics Refs: [35,38,53,91].	Stool (high signal, complex matrix) Mucosal samples Refs: [92,93].	Standardize collection, storage, and metadata (diet/antibiotics). Refs: [36,37,51,94].

BEV, Bacterial Extracellular Vesicle; CD, Cluster of Differentiation (e.g., CD9, CD63, CD81); CRC, Colorectal Cancer; EV, Extracellular Vesicle; lncRNA, long non-coding RNA; LPS, Lipopolysaccharide; LTA, Lipoteichoic Acid; m/lEV, medium/large Extracellular Vesicle; miRNA, microRNA; OMV, Outer Membrane Vesicle; PRR, Pattern Recognition Receptor; sEV, small Extracellular Vesicle; TLR, Toll-like Receptor; TSG101, Tumor Susceptibility Gene 101.

**Table 2:** Candidate vesicular mediators and intersection-layer nodes in CRC cachexia.

Source (EV Class)	Representative Cargo/Ligand	Primary Decoding Hub(s)	Muscle Phenotype (Conceptual)	Evidence Level & Limitations	Translational Opportunities
Tumor/host EVs	miRNAs (e.g., miR-195a-5p, miR-125b-1-3p) Ref: [85]	Bcl-2 pathway (Apoptosis regulation)	Myoblast apoptosis Muscle fiber atrophy	Preclinical (In vivo) Limitation: Human causality remains unproven (serum association only)	Targeting EV uptake miRNA antagonists
Tumor microvesicles	miR-21 (and related miRNAs) Ref: [84]	Endosomal TLR7 signaling Ref: [84]	Myoblast apoptosis Muscle wasting	Preclinical (In vivo) Limitation: Human TLR7 sensing of EV-RNA is debated.	Endotype enrichment RNA-sensing TLR modulation
Tumor exosomes	miRNA programs (Bcl-2 axis) Ref: [85]	Apoptosis regulators Stress pathways	Apoptosis Impaired myogenesis	Preclinical (In vivo) Model-dependent	Target uptake Cargo validation
Tumor-derived exosomes	GDF15 (Predominantly soluble; fractionally EV-associated) Ref: [87]	GFRAL (Brainstem) (Peripheral stress → Central anorexia) Refs: [106,107,108,109]	Appetite suppression Weight loss Indirect muscle effects	Strong Clinical Validation (Axis) Limitation: EV-specific contribution requires proof.	Therapeutic antibody (e.g., ponsegromab) Ref: [110]
Microbial BEVs/OMVs	Gram (−): LPS Gram (+): LTA, Peptidoglycan Refs: [43,44,45,46,47,48]	TLR4 (LPS) TLR2 (LTA) Refs: [77,78,79,80,81]	Inflammation-driven proteolysis Autophagy	Plausible pathway (inferred from sepsis/colitis models) Limitation: Direct muscle causality in human CRC is unproven.	TLR4 blockade Barrier restoration
*F. nucleatum* OMVs	Inflammatory signals Virulence factors Ref: [64]	Mucosal inflammation Immune modulation	Systemic inflammation Indirect muscle wasting	Preclinical/Associative Validated in gut models.	Vesicle-associated metagenomics Ref: [67]
Mixed particle populations	EV–lipoprotein co-isolates (plasma) Refs: [38,53]	Non-specific uptake Confounded readouts	False-positive biomarker signals	Known Methodological Risk Limitation: Major confounder in plasma assays.	Standardize separation Orthogonal validation Refs: [35,36,37,38,51]
Stool EV biomarkers	Fecal EV markers (e.g., CD147, A33) Ref: [93]	Not a decoding hub (Diagnostic readout)	Risk stratification Early detection	Human Cohort Evidence Limitation: Complex matrix & enzymatic degradation.	Develop CRC-cachexia panels Longitudinal validation Ref: [92]

‘Preclinical in vivo support’ indicates that vesicle administration or vesicle/decoding-hub perturbation in animal models produces cachexia-relevant endpoints (e.g., muscle mass/strength, UPS/ALP activation) with vesicle characterization and contamination controls; ‘Preclinical intestinal evidence; human associations’ indicates mechanistic support in intestinal or tumor models plus cohort-level association without direct cachexia causality; ‘Human cohort evidence for CRC detection/prognosis’ indicates clinical association for CRC but not cachexia endpoints; ‘Plausible; direct CRC-cachexia causality unproven’ indicates mechanistic plausibility or pathway linkage without direct in vivo cachexia demonstration; ‘Known methodological risk’ flags frequent confounding (e.g., lipoprotein co-isolation, endotoxin carryover) requiring heightened scrutiny. BEV, Bacterial Extracellular Vesicle; Bcl-2, B-cell lymphoma 2; CRC, Colorectal Cancer; EV, Extracellular Vesicle; GDF15, Growth Differentiation Factor 15; GFRAL, GDNF Family Receptor Alpha-like; LPS, Lipopolysaccharide; LTA, Lipoteichoic Acid; OMV, Outer Membrane Vesicle; PRR, Pattern Recognition Receptor; TLR, Toll-like Receptor.

## Data Availability

The original contributions presented in this study are included in the article. Further inquiries can be directed to the corresponding author(s).

## Supplementary Materials

The following supporting information can be downloaded at https://www.mdpi.com/article/10.3390/cancers18030522/s1, Table S1: Qualitative appraisal template for cross-kingdom EV/BEV studies using Box 1 evidentiary criteria.

## Abbreviations

The following abbreviations are used in this manuscript:

ALP	Autophagy–lysosome pathway
BEVs	Bacterial extracellular vesicles
CRC	Colorectal cancer
EVs	Extracellular vesicles
fEVs	Fecal extracellular vesicles
GDF15	Growth differentiation factor 15
GFRAL	GDNF family receptor alpha-like
HMGB1	High mobility group box 1
Hsp70/90	Heat shock protein 70/90
LPS	Lipopolysaccharide
MISEV	Minimal Information for Studies of Extracellular Vesicles
mEVs	Medium/large extracellular vesicles
OMVs	Outer membrane vesicles
PRRs	Pattern-recognition receptors
sEVs	Small extracellular vesicles
SMI	Skeletal muscle index
TLR	Toll-like receptor
UPS	Ubiquitin–proteasome system
