# Supplementary material for: The Vesicular Intersection Layer: A Framework for Cross-Kingdom Extracellular Vesicle Signaling That May Connect Gut Dysbiosis to Skeletal Muscle Wasting in Colorectal Cancer Cachexia
Source: Cancers (Basel). 2026 Feb 5;18(3):522. doi: 10.3390/cancers18030522 (PMC12896952; doi:10.3390/cancers18030522)
Supplement: Supplementary file 1 [file cancers-18-00522-s001.zip › cancers-4120804-supplementary.pdf]

Supplementary Table S1. Qualitative appraisal template for cross-kingdom EV/BEV studies using Box 1 evidentiary criteria.

Study	Model/cohort	Vesicle source claimed	Attribution (Y/P/N)	Purity controls (Y/P/N)	Cargo localization (Y/P/N)	Endotoxin carryover controls (Y/P/N)	In vivo cachexia endpoints (Y/P/N)	Key confounders addressed	Evidence level note
Miao et al.(2021) Ref: [85]	C26 tumor-bearing mice & CRC patient serum	Tumor-Tumor-derived Exosomes (Tex) carrying miR-195a-5p & miR-125b-1-3pEVs (TEVs)	Y (GW4869 used to block EV release; cell-specific origin confirmed)	Y (TEM, NTA, Western Blot for CD63/TSG101/Calnexin negative control)	Y (RNase A treatment with/without Triton X-100 to prove luminal cargo)	N/A (Sterile tumor cell lines used; endotoxin not a primary confounder)	Y (Muscle weight, fiber CSA, grip strength, apoptotic markers)	Comparison with non-cachectic cell lines; Specific miRNA inhibitors used *in vivo*.	High: Strong causality shown (Blockade of EV release & specific cargo inhibition prevented wasting).
Engevik et al. (2021) Ref: [64]	F. nucleatum culture, Caco-2 cells, & Mice	Bacterial OMVs (Outer Membrane Vesicles)	Y (Purified OMVs from bacterial culture)	Y (TEM, Proteomic analysis)	P (LPS is surface-displayed; specific cargo topology less emphasized than inflammatory effect)	Y (Comparison of OMVs vs. Free LPS effects; Dose-dependent responses)	N (Focuses on intestinal inflammation/barrier disruption, not skeletal muscle endpoints)	Differentiated between whole bacteria and purified OMV effects.	Moderate (Gut-specific): Demonstrates OMV-driven inflammation/barrier loss, but direct link to muscle wasting is inferred.
Zhang et al. (2023) Ref: [93]	*Human CRC Stool samples (Diagnosis/Prognosis cohort)*	Fecal EVs (Host & Microbial mixture)	P (Identified markers CD147, A33; difficult to fully separate host vs. microbial origin)	Y (Ultracentrifugation + Density Gradient; TEM, NTA)	N (Focus on proteomic biomarker profiling)	N/A (Biomarker study; functional assay not performed)	N (Correlated with CRC stage/diagnosis, not specific cachexia phenotypes)	Validated against tissue expression; Large cohort validation.	Low (for mechanism): Clinical association/diagnostic value established, but cachexia causality unproven.

(Note: Y=Yes/Robust, P=Partial/Incomplete, N=No/Absent)

This table provides a structured template to document, in a transparent and reproducible manner, whether individual studies report key elements required to support cross-kingdom vesicle attribution and causal interpretation, including vesicle source attribution, purity controls, cargo localization/topology, endotoxin carryover controls, and in vivo cachexia endpoint anchoring (scored as Y/P/N). "Key confounders addressed" and "Evidence level note" are free-text fields intended to capture major clinical/experimental covariates and to contextualize the overall strength of evidence.

Legend for Y/P/N fields in Supplementary Table S1

Y (Yes) = explicitly performed and sufficiently reported; P (Partial) = addressed but incomplete/indirect; N (No) = not addressed or not reported.

- ❿ Attribution (Y/P/N): Y if vesicle origin is supported by orthogonal evidence (source-specific markers/controls and/or perturbation/labeling consistent with the claimed source); P if based mainly on indirect inference or limited markers; N if source is assumed.

- ❿ Purity controls (Y/P/N): Y if major co-isolates/contaminants and process controls are assessed (e.g., orthogonal separation/QC and negative markers; lipoprotein assessment for plasma as applicable); P if limited QC without key contaminant/process controls; N if not evaluated.
- ❿ Cargo localization (Y/P/N): Y if topology/localization is tested (e.g., nuclease/protease protection ± detergent or equivalent); P if cargo is measured without localization; N if not assessed.
- ❿ Endotoxin carryover controls (Y/P/N): Y if endotoxin is quantified and/or functionally controlled (e.g., polymyxin B/TLR4 inhibition or equivalent) when functional claims are made; P if only partial testing/controls; N if absent.
- ❿ In vivo cachexia endpoints (Y/P/N): Y if in vivo cachexia-relevant outcomes include objective muscle endpoints (mass/CSA/CT indices ± function/food intake) beyond tumor burden; P if limited (e.g., weight only); N if none.

Key confounders are addressed, and Evidence level notes are free-text fields (e.g., stage/tumor burden, chemotherapy/antibiotics, diet, inflammation, sex/age, sampling time points; human longitudinal vs. cross-sectional; animal perturbation vs. correlative; in vitro only).
